# The Proline/Arginine Dipeptide from Hexanucleotide Repeat Expanded *C9ORF72* Inhibits the Proteasome

**DOI:** 10.1523/ENEURO.0249-16.2017

**Published:** 2017-01-31

**Authors:** Rahul Gupta, Matthews Lan, Jelena Mojsilovic-Petrovic, Won Hoon Choi, Nathaniel Safren, Sami Barmada, Min Jae Lee, Robert Kalb

**Affiliations:** 1 Department of Chemical and Biomolecular Engineering, School of Engineering and Applied Sciences, University of Pennsylvania, Philadelphia, PA, 19104; 2 Department of Biology, College of Arts and Sciences, University of Pennsylvania, Philadelphia, PA, 19104; 3 Department of Biochemistry, College of Arts and Sciences, University of Pennsylvania, Philadelphia, PA, 19104; 4 Division of Neurology, Department of Pediatrics, Research Institute, Children’s Hospital of Philadelphia, Philadelphia, PA, 19104; 5 Department of Biochemistry and Molecular Biology, Seoul National University College of Medicine, Seoul 03080, Republic of Korea; 6 Department of Neurology, University of Michigan, Ann Arbor, MI, 48109; 7 Department of Neurology, Perelman School of Medicine, University of Pennsylvania, Philadelphia, PA, 19104

**Keywords:** ALS, frontotemporal dementia, lysosome-autophagy, motor neuron, proteasome

## Abstract

An intronic hexanucleotide repeat expansion (HRE) mutation in the *C9ORF72* gene is the most common cause of familial ALS and frontotemporal dementia (FTD) and is found in ∼7% of individuals with apparently sporadic disease. Several different diamino acid peptides can be generated from the HRE by noncanonical translation (repeat-associated non-ATG translation, or RAN translation), and some of these peptides can be toxic. Here, we studied the effects of two arginine containing RAN translation products [proline/arginine repeated 20 times (PR_20_) and glycine/arginine repeated 20 times (GR_20_)] in primary rat spinal cord neuron cultures grown on an astrocyte feeder layer. We find that PR_20_ kills motor neurons with an LD_50_ of 2 µM, but in contrast to the effects of other ALS-causing mutant proteins (i.e., SOD or TDP43), PR_20_ does not evoke the biochemical signature of mitochondrial dysfunction, ER stress, or mTORC down-regulation. PR_20_ does result in a time-dependent build-up of ubiquitylated substrates, and this is associated with a reduction of flux through both autophagic and proteasomal degradation pathways. GR_20_, however, does not have these effects. The effects of PR_20_ on the proteasome are likely to be direct because (1) PR_20_ physically associates with proteasomes in biochemical assays, and (2) PR_20_ inhibits the degradation of a ubiquitylated test substrate when presented to purified proteasomes. Application of a proteasomal activator (IU1) blocks the toxic effects of PR_20_ on motor neuron survival. This work suggests that proteasomal activators have therapeutic potential in individuals with *C9ORF72* HRE.

## Significance Statement

Peptides made up of two alternating amino acids, proline/arginine (PR) or glycine/arginine (GR), are thought to contribute to the pathophysiology of familial ALS and frontotemporal dementia (FTD) caused by expansion of the intronic microsatellite repeat sequence GGGGCC in the *C9ORF72* gene. Here, we show that proline/arginine repeated 20 times (PR_20_) is toxic to motor neurons and inhibits substrate flux through the proteasome and the lysosomal-autophagy pathway. Stimulation of the proteasome alleviates this toxicity, suggesting that targeting the PR_20_-proteasome interaction may have therapeutic potential.

## Introduction

ALS is an adult-onset, fatal neurodegenerative disease that manifests with progressive weakness, muscle wasting, spasticity, and respiratory failure (Wijesekera and Leigh, 2009), signs and symptoms that result from the death of upper and lower motor neurons (Rowland and Shneider, 2001). Approximately 10% of ALS cases are caused by single gene mutations; the remaining ∼90% of cases are sporadic (Kiernan et al., 2011).

To date, the most common genetic abnormality underlying familial ALS is an expansion of the hexanucleotide sequence GGGGCC in the intron located between exons 1a and 1b of the *C9ORF72* gene (Renton et al., 2011; DeJesus-Hernandez et al., 2011). The majority of normal individuals have fewer than 8 GGGGCC repeats, whereas patients can have hexanucleotide repeat expansions (HREs) consisting of several hundreds or even thousands of repeats (Rutherford et al., 2012). Pre-mRNA transcripts containing the HRE adopt a G-quadruplex structure that can lead to sequestration of RNA-binding proteins and reduced abundance of the mature *C9ORF72* mRNA (Conlon et al., 2016; Lee et al., 2016; Lin et al., 2016). In some experimental platforms, the pathophysiology of *C9ORF72* HRE can at least partially be linked to these mRNA structures (Haeusler et al., 2014).

Additionally, the pre-mRNA produced from the HRE undergoes translation despite the lack of the ATG start codon. This repeat-associated non-ATG (RAN) translation results in the production of 5 different dipeptide repeat (DPR) proteins, depending on the reading frame and on the translation of the sense or antisense strand. The arginine-rich RAN translation products PR_n_ and GR_n_ as well as GA_n_ (where the *n* represents the number of dipeptide repeats) have been shown to result in neurodegeneration (Mizielinska et al., 2014; Zhang et al., 2014). Recent work establishes that DPRs can undergo cell-to-cell transfer (Westergard et al., 2016).

The mechanism by which *C9ORF72*-associated RNA or DPR proteins impart toxicity onto cells is under active investigation. Although recent work points to defects in nucleocytoplasmic shuttling in *C9ORF72* HRE pathophysiology (Freibaum et al., 2015; Joviči et al., 2015; Zhang et al., 2015; Boeynaems et al., 2016), many questions remain. First, is HRE-mediated toxicity due to the HRE mRNA, DPR proteins, or both? Although several groups that have found defects in nucleocytoplasmic transport in the setting of *C9ORF72* HRE, they come to opposite conclusions regarding the toxic agent (Joviči et al., 2015; Freibaum et al., 2015; Zhang et al., 2015). Considering the differences in molecular structure between the HRE mRNA/DNA and DPR proteins, *a priori*, it seems unlikely that both would affect similar structures in the nuclear pore complex. Second, by what mechanism are impairments in nucleocytoplasmic shuttling injurious to cells and why are select neuronal populations affected? Essentially no information is extant on these issues. A system that unambiguously dissociates RNA from DPR protein toxicity could begin to provide insight into these pressing questions.

Here, we explore DPR protein toxicity by interrogating primary spinal cord neuronal cultures grown on an astrocyte feeder layer treated with synthetically produced proline/arginine repeated 20 times (PR_20_) or glycine/arginine repeated 20 times (GR_20_), obviating the distinction between DPR protein and mRNA-mediated toxicity. Unexpectedly, perturbed cellular processes previously implicated in genetic models of ALS (Kong and Xu, 1998; Betz and Hall, 2013; Saxena et al., 2013; Perera et al., 2014), such as mitochondrial dysfunction, ER stress, and mTOR down-regulation, were unaffected by PR_20_ in this model. Instead, we find that PR_20_ (but not GR_20_) disrupts the activity of the ubiquitin-proteasome system (UPS) and the autophagy pathway. In addition, PR_20_ (but not GR_20_) associates with and directly inhibits proteasomes *in vitro*. We show that stimulating UPS activity protects against PR_20_-mediated motor neuron death. The implication of the cellular protein degradation pathway in PR_20_ toxicity suggests that the build-up of cellular proteins that are marked for degradation through ubiquitylation may result in RAN peptide-mediated *C9ORF72* HRE toxicity.

## Materials and Methods

### Antibodies

The following antibodies were used in this study: ubiquitin (Dako Z0458), phospho-P70 S6K (Cell Signaling, T389), P70 S6K (Cell Signaling, 9202S), phospho-4EBP1 (Cell Signaling, 2855S), 4EBP1 (Cell Signaling, 9452), phospho-AMPK (Cell Signaling, 2535S), AMPK (Cell Signaling, 2532S), HA (Roche, 3F10), α3 (Enzo Life Sciences, MCP257), T7 (EMD Millipore, 69522) β-actin (Sigma A2066), anti-mouse and anti-rabbit Alexa Fluor 488 and 594 antibodies (Invitrogen), and anti-mouse and anti-rabbit IRDye antibodies (Li-Cor).

### Culture generation

Mixed spinal cord neuron cultures were prepared as described previously (Mojsilovic-Petrovic et al., 2006). Briefly, an astrocyte feeder layer was prepared from the cortex of newborn Sprague Dawley rat pups [postnatal day 2 (P2)] and grown to ∼80% confluency. Subsequently, dissociated embryonic day 15 (E15) spinal cord neurons were added. One to two days later, AraC (5 mM) (catalog #C6645; Sigma) was added for 24 h to arrest astrocyte proliferation. Cultures were maintained in glia-conditioned medium supplemented with the following trophic factors (1.0 ng/ml each): human neurotrophin-3, human neurotrophin-4, human brain-derived neurotrophic factor, and rat ciliary neurotrophic factor (Alomone Labs). Half of the culture medium was replaced on a biweekly basis.

### Toxicity assays and LD_50_ determination of PR_20_ or MG132

Days *in vitro* (DIV)14 mixed spinal cord cultures were exposed to PR_20_ or GR_20_ by addition of synthetic peptide to the media one time. Cultures were fixed 5 d later with freshly prepared 4% paraformaldehyde for 20 min, washed extensively with 0.1 M PBS, pH 7.4, and immunostained with SMI32 to identify motor neurons. Immunopositive cells >25 µm in diameter are motor neurons based on their costaining for peripherin, ChAT, and islet 1/2 (Mojsilovic-Petrovic et al., 2006). Motor neurons were counted in three nonoverlapping, randomly selected fields on a coverslip using a 5[times objective, and the values from a single coverslip averaged. The results of 3-5 independently treated coverslips were the basis and means and variance used in statistical analysis. Each experiment was repeated with independently generated culture 4+ times (Mojsilovic-Petrovic et al., 2006).

To determine the LD_50_ of DPR peptides, PR_20_ or GR_20_ was added once to the culture media of DIV14 cultures such that the final concentration varied from 2 nM to 35 µM at approximately 10-fold increments. Motor neuron survival 5 d later was determined. Once the approximate LD_50_ was determined, we repeated the analysis with more closely spaced concentrations for a more precise definition of the LD_50_. Determination of the LD_50_ of MG132 was accomplished in an identical manner.

For experiments involving IU1, DIV14 cultures were treated with PR_20_ or its vehicle, and every other day, IU1 (or vehicle) was added to the culture media to achieve a final concentration of 5 µM. At DIV19, cultures were fixed and motor neuron number determined as described above.

For experiments involving astrocytes only, freshly dissociated astrocytes were plated in 96-well dishes and maintained as above. Near confluent cultures were then exposed to various concentrations of PR_20_ or GR_20_ for 5 d and the XTT Cell Viability assay (Thermo Fisher Scientific, catalog #X6493) was performed per manufacturers guidelines. Absorbance at 450 nM was obtained using a BIO-TEK Synergy HT plate reader. This experiment was performed in triplicate.

### PR_20_ treatment for biochemical assays

Confluent dishes of astrocyte neuron cocultures at 37°C had volumes equalized to 2 ml of cell culture media each. Approximately 300 μl of media from each dish was removed and pooled with other dishes, PR_20_ stock solution was added, and the mixture was then added back to the corresponding culture dish, so that each dish contained a final concentration of 2 μM. All plates were incubated with PR_20_ for 48 h.

### Ubiquitylation assay

The ubiquitylation assay involved acute time point treatments with PR_20_. 16 h prior to scheduled lysis, two dishes were treated to a final concentration of 2 μM PR_20_ using the methods described in the PR_20_ treatment for biochemical assays subsection. This was repeated 8 h prior to lysis, 4 h prior to lysis, and 2 h prior to lysis. Samples were then lysed in RIPA buffer as described in Cell lysis for ubiquitin blots and processed for Western blot analysis.

### MG-132 flux assay

MG-132 (Sigma) was used to inhibit proteasomal function. Cells were initially treated with PR_20_ for a total of 48 h using the method outlined in PR_20_ treatment for biochemical assays. Four hours prior to lysis, these cells were treated with MG-132 or vehicle. The MG-132 was kept in a stock solution in DMSO. This was added directly to culture to achieve a final concentration of 5 μM. Cells were then lysed in RIPA buffer and immunoblotted for ubiquitin.

### Cell lysis

Lysis buffer was prepared containing 1% (v/v) protease inhibitor cocktail, 1% (v/v) PMSF, and 1% (v/v) leucine in 1% RIPA buffer solution on ice. Culture dishes were washed once with ice-cold PBS to remove any remaining media. A total of 125 μl (35-mm dish), 150 μl (6-well plate), or 250 μL (60-mm dish) of lysis buffer was added to each plate, and cells were scraped into a cold 1.5-ml tube. Samples were sonicated for 30 s at 20% intensity, placed on a rotator at 4°C for 20 min, and then centrifuged for 15 min at 13,200 rpm. Supernatant was then removed from each tube for further analysis.

### Cell lysis for ubiquitin blots

Ubiquitin analysis requires the use of deubiquitinase (DUB) inhibitors. Lysis without these inhibitors runs the risk of degradation of the substrates to be targeted. As such, *N*-ethylmaleimide (Sigma), a potent DUB inhibitor, was used in the lysis buffer. The lysis buffer was prepared containing 1% (v/v) protease inhibitor cocktail, 1% (v/v) PMSF, 1% (v/v) leucine, and 50 μM NEM in 1% RIPA buffer solution. The remainder of the lysis procedure follows Cell lysis.

### Membrane stripping

Once scanning of the nitrocellulose membranes probed for 4EBP1/P-4EBP1 and S6K/P-S6K had been completed, Restore Western Blot Stripping Buffer (Thermo Fisher Scientific) was used to strip the blot of antibodies. The membranes were washed twice in PBS-T for 10 min each and then placed in the stripping buffer for 22 min. The membranes were then washed twice once again in PBS-T for 10 min. They were then placed in blocking solution (5% w/v milk in PBS-T) for 45 min. This resulted in membranes with sample attached and no antibodies, ready to be probed for actin.

### *In vitro* Ub-Sic1 degradation assays

Polyubiquitylated Sic1 proteins with PY degron motifs and T7 tag (Ub-Sic1) was prepared as previously described by Choi et al. (2016). Different concentrations of PR_20_ peptides (0, 50, or 500 nM) and purified affinity-purified human proteasomes (5 nM) were preincubated 20 min in proteasome assay buffer [50 mM Tris-HCl (pH 7.5), 100 mM NaCl, 10% glycerol, 2 mM ATP, 10 mM MgCl_2_, and 1 mM DTT] on ice, and 20 nM reconstituted Ub-Sic1 was subsequently added. Degradation of Ub-Sic1 and PR_20_ peptides was monitored by immunoblotting using anti-T7 and anti-HA antibodies, respectively. Band intensities were quantified using ImageJ software (version 1.48k, NIH) from three independent immunoblotting assays (*n* = 3).

### Direct interaction between PR_20_ and human proteasomes

Whole-cell extracts from a stable HEK293T cell line harboring HTBH-tagged β4 subunits were prepared as in Han et al. (2014) and incubated with HA-tagged PR_20_ or GR_20_ peptides (2 µM) 2 h at 4^o^C. The resulting proteasome-dipeptide complexes were pull-downed with streptavidin agarose beads (Millipore) for 3 h at 4^o^C. Unbound proteasomes in the supernatants were discarded, and the pellets were mixed with 2× SDS sample buffer. Different amounts of samples were resolved by SDS-PAGE/immunoblotting against HA and a proteasome core particle α3 subunit.

### Autophagic flux assay

Primary spinal neurons were dissected from E15 rat pups and cultured at a density of 2.5 × 10^5^ cells/ml on a laminin/poly-lysine-coated 96-well plate in motor neuron media. Four days after plating, neurons were transfected with Lipofectamine 2000 (Invitrogen). DNA-Lipofectamine mixtures were incubated with neurons for 20 min followed by rinsing. Neurons were then placed in 100 µL of conditioned media that was collected immediately prior to transfection and half fresh motor neuron media containing 4 µM PR_20_, resulting in a final concentration of 2 µM PR_20_.

Optical pulse labeling experiments were performed using an automated microscopy platform previously described by Arrasate et al. (2004) and Barmada et al. (2014). A Nikon Eclipse Ti inverted microscope equipped with a PerfectFocus system, 20× objective lens and an Andor iXon3 897 EMCCD camera were used to acquire images. Samples were illuminated with a Sutter Instruments Lamba XL lamp. The microscope and related components were encased in custom-built plexiglass enclosure set to maintain the temperature at 37°C and CO_2_ at 5%. Semrock GFP and TRITC filters were used for excitation and detection. A Semrock 405-nm long-pass filter was used for photoactivation. Image acquisition and stage movements were accomplished in an automated fashion using µManager and original code written in BeanShell. For all experiments, photoconversion occurred 24 h following transfection. GFP and TRITC images were acquired immediately following activation and subsequently every 2 h for 12 h. Images were acquired in a similar 12-h window starting 48 h after PR_20_ treatment. Using in-house image analysis scripts, neuronal cell bodies were identified based on their fluorescent intensity and morphology, and a ROI was drawn around the perimeter of each cell at each time. Single-cell TRITC intensity values were recorded and used to fit a first order exponential decay curve for individual neurons and calculate a Dendra2-light chain 3 (LC3) half-life for each cell.

### Statistical analysis

To determine whether IU1 treatment had an effect on cell survival, a one tailed, one sample Student’s *t* test was used to test the hypothesis that IU1 would improve cell survival when used with PR_20_.

To test for group differences in the flux assay, a permutation test was used. This is a nonparametric statistical test that was implemented in MATLAB. The goal of the test was to determine whether the +MG132/−MG132 ratio changed significantly in the presence of PR_20_. The test first quantifies this by taking the average of each experimental group and then finding the difference of ratios. It then permutes two samples located in two groups randomly, and determines that difference between averages again. This is repeated 50,000 times, and a histogram is plotted. This histogram represents the distribution of random noise in the sample. Since a higher difference in the +MG132/−MG132 ratio is a more “significant” result, the percentage of the random noise produced from 50,000 permutations that is higher in magnitude than the true difference (calculated from un-permuted data) is the *p* value.

Permutations of a data point in a +MG132 into a group labeled −MG132 (or vice versa) were not allowed. This is because these permutations would produce an obviously significant result, as MG132 is a known proteasomal inhibitor that significantly increases ubiquitin levels in the cell. Thus, this group difference was not interesting to us.

To assess group differences in the autophagic flux assay, the two-sided Kolmogorov–Smirnov test was used. This nonparametric test determines the probability that two continuous, one-dimensional probability distributions are drawn from the same distribution or not.

## Results

### PR_20_ added to media is taken up by neurons and results in death

**Figure 1:**
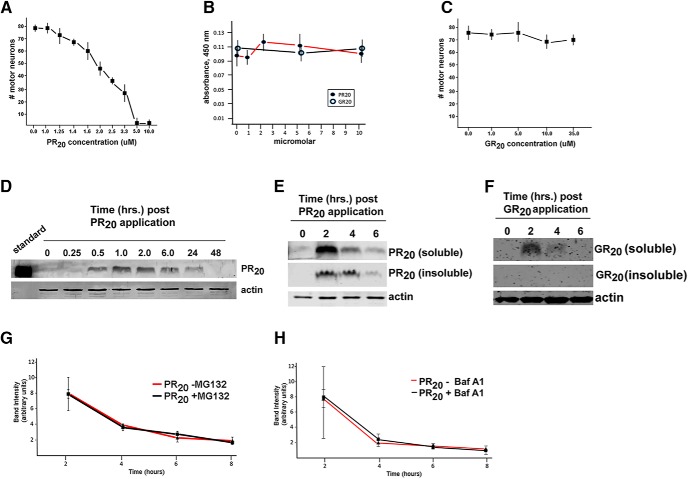
. PR_20_, but not GR_20_, is toxic to motor neurons: kinetics of cellular accumulation and disposal. Mixed spinal cord neuron cultures or pure astrocyte cultures were exposed to PR_20_- or GR_20_-containing media; survival assays and biochemical interrogations followed. ***A***, The survival of motor neurons was determined 5 d after exposure to various concentrations of PR_20_. The LD_50_ of PR_20_ is ∼ 2 µM. ***B***, The survival of astrocytes was determined using the colorimetric XTT assay 5 d after exposure to various concentrations of PR_20_ or GR_20_. No death was seen up to 10 µM DPR. ***C***, The survival of motor neurons was determined 5 d after exposure to various concentrations of GR_20_. No death was seen up to 35 µM GR_20_. ***D***, Immunoblotting for HA-tagged PR_20_ at various time points after addition of 2 µM PR_20_ to mixed spinal cord cultures. PR_20_ is first detectable in cell lysates approximately 0.5 h after exposure and rises to a maximum at 1.0-2.0 h. The Western blot signal declines thereafter and is undetectable at the 48-h time point. ***E***, In a 2-h pulse-chase paradigm after 2 µM PR_20_ application, Western blot signal is detectable in the soluble and insoluble fractions at the 2-h time point, and the signal diminishes thereafter to a barely detectable level by the 6-h time point. ***F***, In a 2-h pulse-chase paradigm after 10 µM GR_20_ application, Western blot signal is detectable in the soluble fraction only at the 2-h time point, and the signal diminishes thereafter to a barely detectable level by the 4-h time point. ***G***, Mixed spinal cord cultures were pulsed for 2 h with 2 µM PR_20_ and the chase media contained MG132 (5 µM) or vehicle. Cell lysates were prepared at time intervals thereafter, and quantitative image analysis of the resultant Western blots showed that the decrement in PR_20_ abundance was the same in the +MG132 condition in comparison with the −MG132 condition. ***H***, Mixed spinal cord cultures were pulsed for 2 h with 2 µM PR_20_, and the chase media contained bafilomycin A1 (Baf A1) (400 nM). Cell lysates were prepared at time intervals thereafter, and quantitative image analysis of the resultant Western blots showed that the decrement in PR_20_ abundance was the same in the +Baf A1 condition in comparison with the –Baf A1 condition.

The difficulty of distinguishing between toxic HRE mRNA and DPR proteins has impeded our understanding of the mechanism underlying *C9ORF72* HRE pathophysiology. As reported in *Science* (Kwon et al., 2014), the McKnight lab devised a strategy to overcome this problem: they studied U2OS cells and human astrocytes directly exposed to synthetic DPR proteins. They found that HA-tagged DPR proteins are taken up by these cells and evoke a variety of biochemical changes. PR_20_ and GR_20_ were shown to be toxic in a concentration range of 10-30 µM depending on the specific assay. We built upon this approach by applying synthetic, HA-tagged, PR_20_ or GR_20_ dipeptides to cultures of rat spinal cord neurons grown on an astrocyte feeder layer, referred to as mixed spinal cord cultures hereafter. We find that PR_20_ is toxic to cells: 5 d after a single application of PR_20_, we observe the death of motor neurons with a LD_50_ of 2 µM (Fig. 1*A*). No significant motor neuron death was seen with 2 µM PR_20_ at the 48-h time point. In pure astrocyte cultures, we found no cell death when PR_20_ was applied up to 10 µM (Fig. 1B). In this assay, GR_20_ was neither toxic to motor neurons when administered up to 35 µM (Fig. 1*C*) nor astrocytes when administered up to 10 µM (Fig. 1*B*).

Kwon et al. (2014) show that 30 min after application to U2OS cells or human astrocytes, PR_20_ accumulates in the nucleus, colocalizing with the nucleolar protein fibrillin. This demonstrates that cells can take up extracellular DPR proteins, which is consistent with the recent demonstration of cell-to-cell transfer of DPRs *in vitro* (Westergard et al., 2016). We investigated the fate of PR_20_ in mixed spinal cord cultures through immunoblotting and immunocytochemistry. After application of 2 µM PR_20_, the immunoblot signal is first detected after 30 min and is maximal at the 1- to 2-h time points (Fig. 1*D*). Despite the continual presence of PR_20_ in the media, the immunoblot signal diminishes over time and is undetectable at the 48-h time point. To gain further insight into the kinetics of the observed decrease in PR_20_ levels in cell lysates over time, we pulsed cells with 2 µM PR_20_ for 2 h, washed, replaced with fresh media (lacking PR_20_), and made cell lysates at regular intervals thereafter. Upon probing for PR_20_ in the soluble and insoluble fractions of cell lysates, we saw maximal immunoreactivity at the 2-h time point and a rapid decline in soluble and insoluble PR_20_ over the subsequent 4 h (Fig. 1*E*). After a single 2-h application of 2 µM GR_20_, the peptide is weakly detectable by immunoblotting in mixed spinal cord lysates. To more easily follow GR_20_ by immunoblotting, we applied 10 µM GR_20_ to cultures and performed the same kinetic analysis as described in Figure 1*D*. In this assay system, GR_20_ is detectable in cell lysates as early as 15 min and maximally accumulates in cells over the first hour of exposure. GR_20_ immunoblot signal falls off rapidly thereafter and is undetectable at the 24-h time point. In the pulse-chase assay, GR_20_ is maximal at the 2-h time point (immediately after the removal of GR_20_ containing media) and is rapidly cleared from the soluble fraction of cells over the next 2-4 h (Fig. 1*F*). GR_20_ is not detectable in the insoluble fraction under these assay conditions. To determine whether the proteasome was involved in the decrease in PR_20_ abundance, we compared the kinetics of the PR_20_ signal when MG132 or vehicle was included in the chase media. The loss of PR_20_ was indistinguishable under these two conditions (Fig. 1*G*). Parallel studies using bafilomycin A1 in the chase media indicated that the lysosomal-autophagy system was similarly uninvolved in the decrease abundance in PR20 over time (Fig. 1*H*).

**Figure 2.**
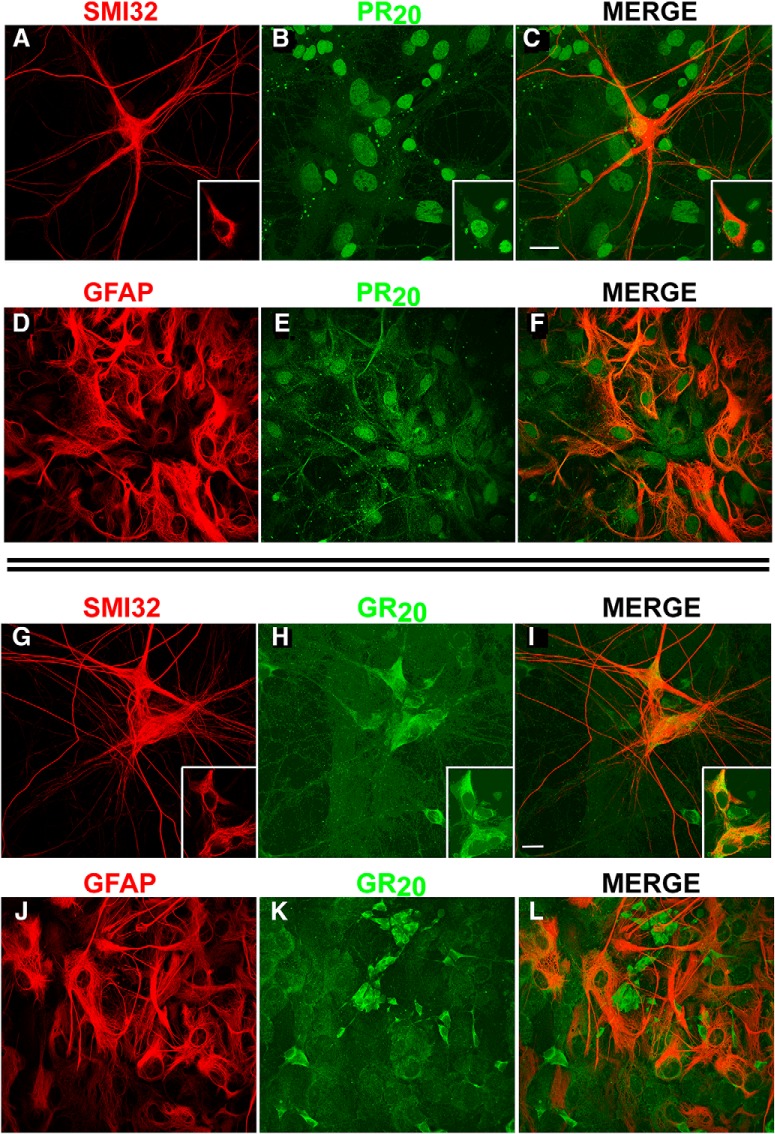
PR_20_ and GR_20_ accumulate in distinct subcellular locations and distinct cell populations. Mixed spinal cord cultures were exposed to 2 µM PR_20_ or GR_20_ for 48 h, fixed, and processed for immunocytochemistry. ***A***, Staining with SMI32 reveals large multipolar motor neurons. The inset shows a single 0.5-µm slice from the confocal data at the level of the nucleus. ***B***, The same field as in **A**, stained for HA-PR_20_ reveals nuclear staining as well as scattered puncta. The inset shows a single 0.5-µm slice at the same level as in (**A**), suggesting nuclear PR_20_ in the motor neuron. ***C***, A merge image of ***A*** and ***B*** reveals nuclear PR_20_ in multiple cells, including the labeled motor neuron. The inset shows the same single 0.5-µm slice from ***A*** and ***B*** unambiguously demonstrating PR_20_ immunoreactivity in motor neurons. Calibration bar = 35 µm. ***D***, Staining for GFAP reveals abundant astrocytes in these cultures. ***E***, The same field as in ***D***, stained for HA-PR_20_ reveals nuclear staining as well as scattered puncta. ***F***, A merge image of ***D*** and ***E*** reveals nuclear PR_20_ in astrocytes. ***G***, Mixed spinal cord cultures were exposed to 2 µM GR_20_ for 48 h, fixed, and processed for immunocytochemistry. Staining with SMI32 reveals large multipolar motor neurons. Inset shows a single 0.5-µm slice from the confocal data at the level of the nucleus. ***H***, The same field as in ***G***, stained for HA-GR_20_ reveals cytoplasmic staining. Inset show a single 0.5-µm slice at the same level as in ***G***, suggesting cytoplasmic GR_20_ in the motor neuron. ***I***, A merge image of ***G*** and ***H*** reveals cytoplasmic GR_20_ in multiple cells, including the labeled motor neurons. Inset shows the same single 0.5-µm slice from the ***G*** and ***H*** unambiguously demonstrating GR_20_ immunoreactivity in motor neurons. Calibration bar = 30 µm. ***J***, Staining for GFAP reveals abundant astrocytes in these cultures. ***K***, The same field as in ***J*** stained for HA-GR_20_ reveals cytoplasmic staining. ***L***, A merge image of ***J*** and ***K*** reveals cytoplasmic GR_20_ is not present in astrocytes.

Immunocytochemical examination at the 2-h time point reveals nuclear PR_20_ immunoreactivity in motor neurons [identified by colocalization with a validated motor neuron marker, SMI32 (Mojsilovic-Petrovic et al., 2006); Fig. 2*A--C*] and other cells including astrocytes (Fig. 2*D--F*). Irregularly-shaped punctate accumulations of PR_20_ immunoreactivity are also visible; some appear to be intracellular and others extracellular (best seen in Figure 2*B,E*). Immunocytochemical examination of mixed spinal cord cultures exposed to 2 µM GR_20_ for 2 h reveals cytosolic GR_20_ immunoreactivity in motor neurons and other cells (Fig. 2*G--I*), but not astrocytes (Fig. 2*J-**-L*).

We draw a number of conclusions from these experiments. First, PR_20_ is toxic to motor neurons with an LD_50_ of 2.0 µM, a value that is 25% of the LD_50_ of 8.4 µM reported by Kwon et al. (2014) that kills human astrocytes. We do not see the astrocytic DPR toxicity which we attribute to procedural differences: (1) Kwon et al. used human astrocytes, whereas we used freshly prepared rat astrocytes; and (2) we applied DPRs once, whereas Kwon et al. did so repeatedly.

Second, when cells contain the maximal amount of PR_20_ (approximately 2 h after treatment), we observe that it accumulates in the nuclei of neurons and astrocytes. As such, PR_20_ acting directly on neurons may be noxious and/or a sublethal insult to astrocytes might be contributing to motor neuron death. Although punctate accumulations of PR_20_, as reported previously (Wen et al., 2014), are also visible, the extent to which nuclear or punctate PR_20_ confers toxicity is not known.

Third, even at a ∼20 times higher concentration, GR_20_ is not toxic to motor neurons. When cells contain the maximal amount of GR_20_ (approximately 2 h after treatment), we find that it selectively accumulates in the cytosol of neurons only. Possible explanations for the benignity of GR_20_ in our system are that the nucleus is the site of DPR protein toxicity and/or that astrocytes contributor to motor neuron injury. The absence of GR_20_ in either or both of these locales apparently renders motor neurons largely insensitive to GR_20_.

Fourth, PR_20_ accumulates to a higher degree and for a longer time than GR_20_ in cells, although the half-life of these dipeptides is similar (on the orders of hours). Neither the ubiquitin-proteasome nor the lysosome-autophagy pathway is likely to be responsible for the time-dependent loss of PR_20_. Owing to how we crafted the sensitivity of Western blotting for DPR proteins, we are probably underestimating the relative abundance of these proteins in our cell lysates. Thus, we suspect that DPR proteins are not completely eliminated from cells within 48 h. It is noteworthy that HA-tagged PR_20_ and GR_20_ have similar molecular weights (6.1 vs 5.3 kDa, respectively) and identical calculated isoelectric point (e.g., pI = 12.37). This suggests that the biological effects of small basic peptides are sequence dependent, implying that they interact with distinct intracellular molecules.

### PR_20_ does not significantly impact mTORC, ER stress, or mitochondrial dysfunction

Cell and animal models of ALS based on the expression of mutant proteins that cause familial ALS (e.g., mTDP43, mSOD1) have implicated reduced mTORC activity, mitochondrial dysfunction, and ER stress (Kong and Xu, 1998; Lim et al., 2012; Betz and Hall, 2013; Saxena et al., 2013; Perera et al., 2014) in the pathophysiology. To understand the mechanism underlying DPR protein toxicity, we asked whether PR_20_ evoked similar biochemical changes in cells.

**Figure 3.**
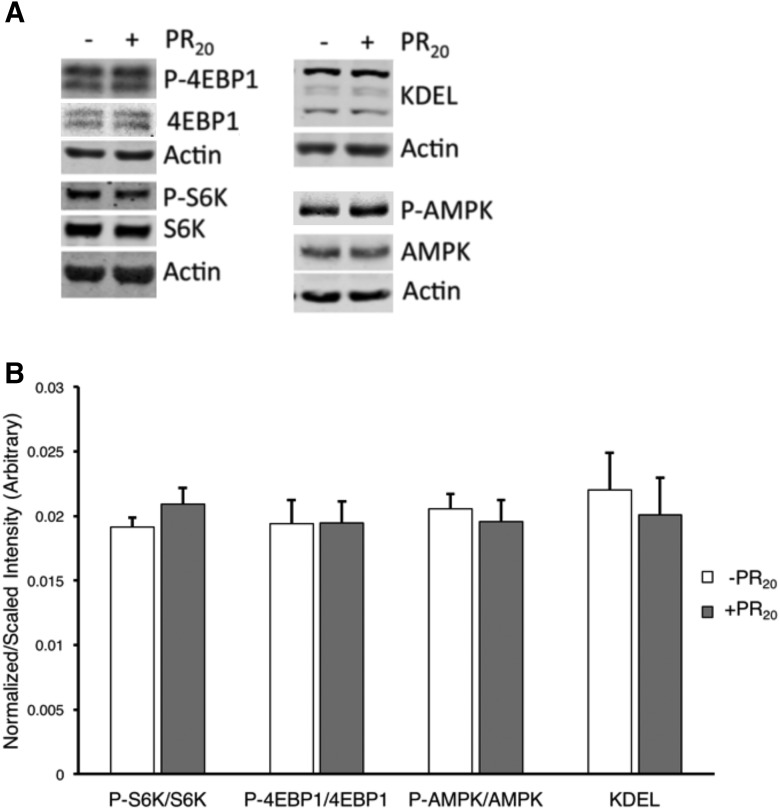
PR_20_ neither influences the activation of TORC or AMPK nor ER stress. Mixed spinal cord cultures were exposed to PR_20_ for 48 h and then processed for immunoblotting. ***A***, Representative immunoblot images of biochemical markers of TORC activation (e.g., phospho-4EBP1 and phospho-S6K), AMPK activation, or activation of the ER stress response (KDEL) shows no difference between PR_20_ versus vehicle-treated cells. ***B***, Quantification of the bands with intensity values from samples were averaged and normalized to actin loading controls. Dark grey bars correspond to the presence of PR_20_, and light grey bars correspond to levels without PR_20_. Error bars represent SE.

Upon treating cultures with 2 µM PR_20_ for 48 h, we used immunoblotting techniques to probe for these biochemical changes (Fig. 3*A,B*). We see no statistically significant differences in the activation level of the mTOR pathway (monitored by the abundance of phosphorylated S6K and 4EBP1), mitochondrial dysfunction (monitored by the abundance of phosphorylated AMPK), or ER stress (monitored by the abundance of KDEL-tagged chaperones BiP and PDI). Biochemical interrogations at earlier time points similarly show no effect of PR_20_ on these pathways (data not shown). These observations suggest that in this experimental paradigm, PR_20_ does not influence pathways previously implicated in mTDP43 and mSOD toxicity. One caveat here is that our culture system contains astrocytes, motor neurons, and other neurons and biochemical changes within a subpopulation of cells might be obscured when we generate cell lysates from this heterogeneous cell population. The cost of using maximally healthy astrocyte/neuronal cocultures that approximate the *in vivo* complexity of cell-cell interactions is the difficulty of studying the biochemistry of a distinct subtype of neuron.

### PR_20_ induces cytoplasmic degradation dysfunction through diminished proteasomal flux

**Figure 4.**
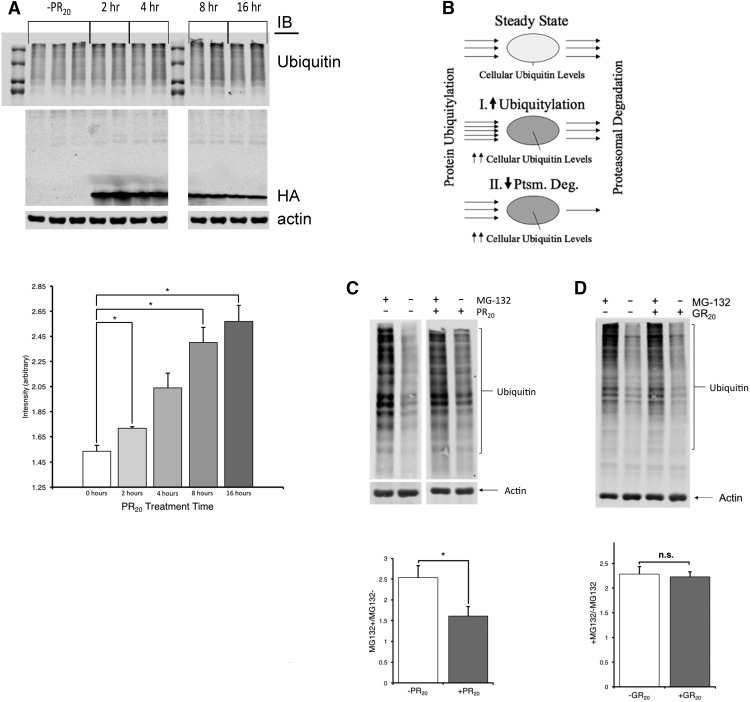
Total ubiquitin levels rise over time in the presence of PR_20_; PR_20_, but not GR_20_, inhibits substrate flux through the ubiquitin-proteasomal system. Mixed spinal cord cultures were exposed to PR_20_ or GR_20_ and subjected to biochemical interrogations. ***A***, Duplicate or triplicate cell lysate samples probed for ubiquitin in the absence of PR_20_ (-PR_20_) or at 2, 4, 8, or 16 h after PR_20_ application. A progressive build-up in total ubiquitin levels occurs over time. The panel below shows quantification of these data with total ubiquitin levels normalized to actin loading controls. Error bars represent SE. Blotting for HA-tagged PR_20_ shows that DPR is present in the lysates but does not migrate as a high molecular weight species as would be expected if PR_20_ was ubiquitylated. Equal amounts of total protein are present in each lane, as reported by the actin blot. ***B***, Cartoon describing two different mechanisms for increased steady state ubiquitin levels: enhanced ubiquitylation versus decreased proteasomal degradation. ***C***, Total ubiquitin levels from cells treated with PR_20_ or vehicle for 48 h and then MG132 or vehicle for 4 h. The difference between the +/- MG132 lanes represents the flux of ubiquitylated substrates over 4 h. Representative ubiquitin blots demonstrate a smaller difference between the +/- MG132 lanes in PR_20_-treated cultures versus vehicle-treated cultures. The permutation test was applied to determine whether statistically significant group differences exist, and the results are shown in the lower panel. There is a reduced flux of ubiquitylated substrates in the PR_20_-treated cells versus vehicle-treated cells. Six independent biological replicates were tested for each condition. *, *p* < 0.05 by the permutation test. ***D***, Representative ubiquitin blots demonstrate no difference between the +/- MG132 lanes in GR_20_-treated cultures versus vehicle-treated cultures. The permutation test was applied to determine whether statistically significant group differences exist, and the results are shown in the lower panel. No group differences were found, and at least 10 independent biological replicates were tested for each condition. n.s., not significant.

A variety of disease-causing proteins can cause UPS inhibition (Bence et al., 2001). Thus, we asked whether PR_20_ influences the ubiquitylation status of the proteome over time. Upon immunoblotting for total cellular ubiquitin at various time points after the application of PR_20_ (vs vehicle) to cultures, we find that PR_20_ treatment is associated with a progressive increase in total ubiquitylated protein levels. This is statistically significant at the 2-, 8-, and 16-h time points (Fig. 4*A*). We considered the possibility that the smear of ubiquitin immunoreactivity might contain PR_20_ immunoreactivity. When we performed immunoblots for HA in these samples, we saw all the PR_20_ immunoreactivity ran at ∼ 10 kDa, the molecular weight we see of pure peptide (Fig. 4*A*.). Thus, this assay provides no evidence that PR_20_ is ubiquitylated.

The increase in the steady-state amount of ubiquitin over time could be attributed to an increase in the rate of ubiquitylation or a decrease in the rate of degradation of ubiquitylated substrates (Fig. 4*B*). This is analogous to the autophagy protein LC3-II whose increased abundance in cells under certain conditions can be attributed to an increase in the rate of production or a decrease in the rate of degradation (which reflects increased or decreased flux through the lysosomal-autophagy pathway, respectively). Although several approaches can be taken to resolve the mechanism, in the autophagy field, the turnover assay is most widely used: the difference in LC3-II levels between samples with or without an autophagy inhibitor (i.e., chloroquine or bafilomycin A1) is used to report flux (Mizushima et al., 2010; Klionsky et al., 2012). We have taken a similar approach here using MG-132, a potent proteasomal degradation inhibitor (Goldberg, 2012). The difference in the quantity of ubiquitylated proteins from cells treated with MG-132 for 4 h versus vehicle reflects the size of the ubiquitylated protein pool normally degraded by the UPS. The larger the difference, the more proteins are degraded. Thus, this is a measure of flux through the UPS.

Parallel sets of mixed cultures were treated with PR_20_ or vehicle for 48 h, and subsequent experimental groups were treated with MG-132 or vehicle for 4 h. Upon lysis, whole-cell ubiquitin levels were determined through immunoblotting. The average of these was taken for the cases with and without PR_20_. A two-tailed permutation test was performed with 50,000 iterations, and statistically significant (*p* < 0.05) group differences were found; proteasomal flux was reduced in the presence of PR_20_ (Fig. 4*C*). In parallel, we examined the effect of GR_20_ on total ubiquitin levels with or without MG-132. GR_20_ neither influenced total ubiquitin levels nor proteasomal flux (Fig. 4*D*). Together with the finding of elevated total ubiquitylated protein levels in PR_20_-treated (but not GR_20_-treated) cultures over time, these observations suggest that PR_20_ specifically acts as a proteasomal inhibitor.

We wondered how soon after application to cultures did PR_20_ inhibit proteasomal flux. Specifically, at the time point when PR_20_ appears maximally within cells (i.e., 2 h; see Figure 1*D*), was proteasomal flux impaired? We repeated the ±MG132 flux assay after 2 h of PR_20_ exposure and found no statistically significance between the PR_20_ versus vehicle-treated cells by permutation test (*n* = 6 per experimental group, 4 experimental groups, *p* = 0.38). This observation suggest that PR_20_ accumulation in cells does not instantaneously lead to proteasomal inhibition. We suspect that the lag between PR_20_ internalization and proteasomal inhibition is an “access” issue, and perhaps this is linked to ephemeral sequestration of DPR proteins in nonmembrane delimited compartments as recently demonstrated by the Taylor and McKnight labs (Lee et al., 2016; Lin et al., 2016). We did not look at time points later than 48 h, because we feared cell morbidity and mortality could have secondary effects on proteasomal function and thus lead to interpretative difficulties.

Since all of these biochemical experiments were performed on mixed spinal cord cultures, it is possible that the effects of PR_20_ are more or less robust within a specific subpopulation of cells. Although the simplest conclusion is that all cells taking up PR_20_ are manifesting the same biochemical phenotype, deployment of cell-type-specific reporters of UPS flux could reveal differences otherwise obscured by our Western blots.

### Autophagic flux is reduced in the presence of PR_20_

**Figure 5.**
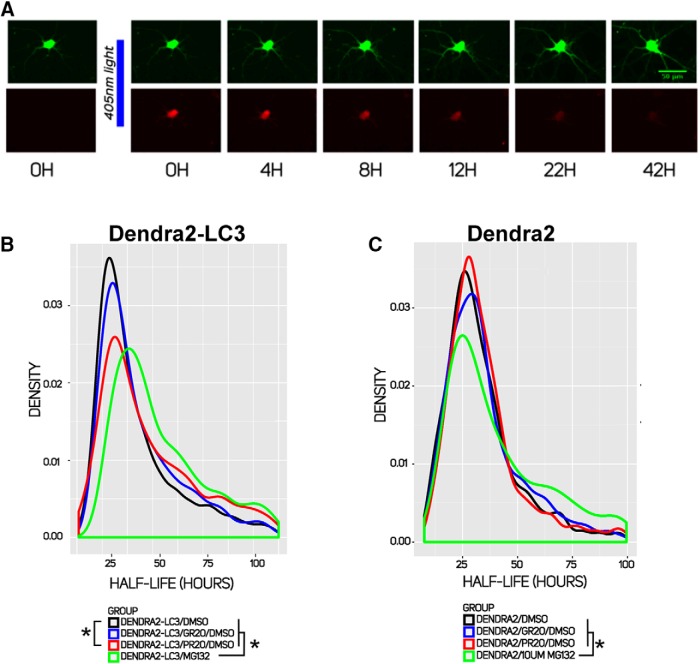
PR_20_ inhibits flux through the lysosomal-autophagy system. *A*, Optical pulse labeling of spinal neurons using automated fluorescent microscopy. Following a pulse of 405-nm light, a portion of Dendra2-LC3 is photoconverted such that its peak emission is red-shifted to 573 nm. By measuring the decay in intensity using a TRITC filter, half-life can be determined in individual neurons. The half-life of Dendra2-LC3 is a measurement of autophagosome turnover, with an increased half-life indicating reduced turnover. ***B***, Distribution of Dendra2-LC3 half-lives in cells treated with 2 µM PR_20_, GR_20_, and 10 µM MG132 compared with those treated with vehicle. * indicates *p* < .05 with the two-sided Kolmogorov–Smirnov (KS) test (GR_20_ *p* = 0.0038, PR_20_ *p* = 2.2 × 10^−16^, MG132 *p* = 2.2 × 10^−16^). ***C***, MG132 significantly extends Dendra2 half-life while 2 µM PR_20_ and GR_20_ do not (MG132 *p* = 3.23 × 10^−5^, two-sided KS test).

We next asked whether PR_20_ influences flux through the lysosomal-macroautophagy pathway (referred to as autophagy hereafter) as well. As mentioned above, one commonly used biochemical approach is to monitor changes in the amount of 1A/1B-LC3, a microtubule-associated protein that acts as both an autophagy marker and substrate, in the presence or absence of an autophagy inhibitor (Mizushima et al., 2010; Klionsky et al., 2016). Unfortunately, this approach is not well suited to investigations using mixed cultures because of the difficulty of resolving the active, lipidated form of LC3, termed LC3-II, from the unmodified LC3-I using immunoblot techniques (Zhai et al., 2015). Moreover, traditional immunoblotting and immunocytochemical techniques can be insensitive and are often difficult to accurately quantify. To overcome these problems, we adopted an optical pulse labeling approach (Tsvetkov et al., 2013; Barmada et al., 2014) to monitor autophagic flux. In this technique, a Dendra2-LC3 fusion protein is introduced into cells. Upon exposure to 405-nm light, the excitation and emission spectra of Dendra2 are irreversibly red shifted. Because LC3 is an autophagy substrate, the rate of Dendra2-LC3 degradation (estimated by the time-dependent loss of red fluorescence) represents a measure of autophagic flux (Koga et al., 2011; Tsvetkov et al., 2013; Barmada et al., 2014; Loos et al., 2014). An example of this phenomenon is shown in Figure 5*A*.

Primary spinal neurons were transfected with a plasmid encoding Dendra2-LC3, and subject to optical pulse labeling using automated longitudinal fluorescence microscopy (Barmada et al., 2014). The half-life of Dendra2-LC3 was measured in thousands of primary spinal neurons on single-cell basis. Four experiment groups were investigated: (1) Dendra2-LC3 + vehicle; (2) Dendra2-LC3 + GR_20_; (3) Dendra2-LC3 + PR_20_; and (4) Dendra2-LC3 + MG132. We observed a wide distribution of neuronal Dendra2-LC3 half-lives in all groups (Fig. 5*B*), consistent with previous studies (Barmada et al., 2014). Treatment with 2 µM PR_20_ peptide led to a significant shift in the population distribution, with a reduction in the peak around ∼20 h and a longer tail of half-lives Dendra2-LC3 (Fig. 5*B*). Compared with the control, treatment with PR_20_ extended the half-life of Dendra2-LC3 by ∼5.6 h (*p* = 2 × 10^−16^). Compared with the control, treatment with GR_20_ to increase the half-life of Dendra2-LC3 by 1.5 h (*p* = 0.0038). Finally, compared with the control, MG132 led to a more marked rightward shift of the curve, and this treatment extended the half-life by 11.9 h (*p* = 2.2 × 10^−16^).

Next, we looked at the distribution of Dendra2 half-lives operating under the presumption that this soluble cytosolic protein is likely to be degraded by the UPS (Fig. 5*C*). Four experiment groups were investigated: (1) Dendra2 + vehicle, (2) Dendra2 + GR_20_, (3) Dendra2 + PR_20_, and (4) Dendra2 + MG132. Compared with the vehicle, MG132 treatment led to a marked rightward shift of the curve and the difference between the Dendra2 half-life in MG132 versus vehicle-treated cells (32.3 vs 29.6 h) was statistically significant (*p* = 3.23 × 10^−5^). Dendra2 half-life in PR_20_- or GR_20_-treated cultures (30.5 and 31.0 h, respectively) did not differ in a statistically significant manner from vehicle-treated cells.

We draw a number of conclusions from these results. First, PR_20_ (and to a far lesser extent GR_20_) retards flux through the autophagy pathway. Second, to the degree that Dendra2 is degraded by the UPS, PR_20_ and GR_20_ had no statistically significant effect. Together, these observations suggest that PR_20_ acts in a relatively selective manner to slow flux through the autophagy pathway. Third, inhibition of the proteasome impairs autophagic flux. This was an unexpected observation, since several prior studies using cell lines have reported autophagy induction upon UPS inhibition (Ding et al., 2007; Lan et al., 2015; Bao et al., 2016). Our findings in neurons may indicate a cell-type-specific relationship between degradative pathways. Alternatively, a nonspecific toxicity to the autophagy pathway may occur when cell function is impaired by UPS blockage. Future work will be required to understand this effect of MG132 on autophagy in neurons.

### PR_20_ imparts direct inhibitory effects on the proteasome *in vitro*

**Figure 6.**
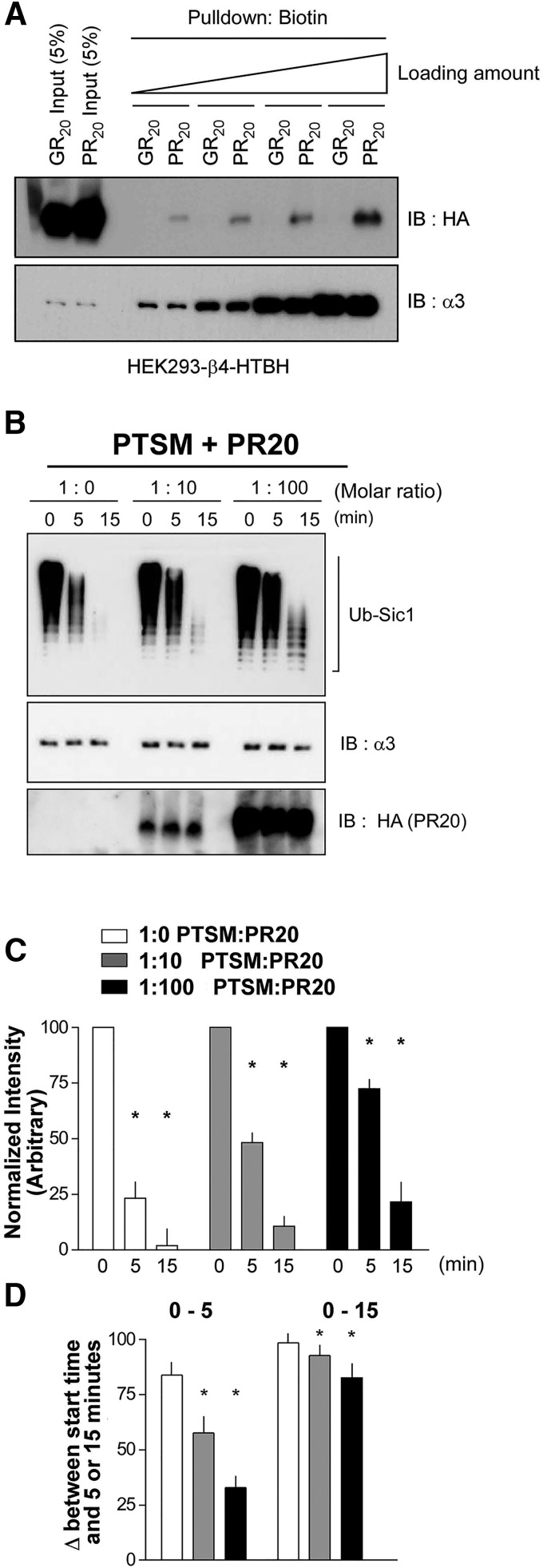
PR_20_, but not GR_20_ binds to proteasomes; PR_20_ inhibits the proteasomal degradation of a test substrate. ***A***, By pulling down human proteasomes and then immunoblotting against HA, a direct association of PR_20_ (but not GR_20_) peptides to the proteasome is demonstrated. Increasing amounts of proteasome leads to more associated PR_20_. Proteasomes are identified using an antibody recognizing the α3 subunit. ***B***, Immunoblot images of Ub-Sic1, HA tag, and α3 loading controls. Molar ratios represent the ratio between 26s proteasomes (PTSM) and PR_20_ peptides. The molar ratios of 1:0, 1:10, and 1:100 were each subject to proteasomal degradation of Ub-Sic1 for 0, 5, and 15 min. Time-dependent loss of Ub-Sic1 is noted. There is no change in the abundance of HA-tagged PR_20_ or the α3 subunit. ***C***, Quantification of Ub-Sic1 levels normalized to loading controls for each condition. Values represent means (*n* = 3), and the error bars represent SE. ***D***, Quantification of the difference between starting level of Ub-Sic1 and Ub-Sic1 abundance at the 5- and 15-min time points. *, *p* < 0.01.

Considering the major role of the proteasome in the degradation of ubiquitylated substrates, we focused on the effects of PR_20_ on the UPS. PR_20_ could impede UPS flux in a variety of ways, such as: (1) reducing access of ubiquitylated substrates to the proteasome and their proper presentation by shuttle factors, (2) increasing de-ubiquitylation, (3) impeding transiting of substrates into the catalytic core of the proteasome, or (4) inhibiting proteolysis. To help distinguish among these possibilities, we began by asking whether PR_20_ directly associates with proteasomes. Cell lysates from HEK293T cells engineered to express biotinylated proteasomes were incubated with PR_20_ or GR_20_. Avidin-coated beads were then used to purify proteasomes, and the pulldown material was subjected to immunoblotting. We find that PR_20_, but not GR_20_, associates with proteasomes in this assay and increasing the amounts of proteasomes leads to more pulldown of PR_20_ (Fig. 6*A*). In light of this observation, we hypothesized that PR_20_ directly inhibits proteasomal substrate degradation.

To investigate the mechanism of action of PR_20_ on the proteasome, we turned to an *in vitro* system with predefined components. Incubation of purified proteasomes with a test substrate (Ub-Sic1) leads to time- and ATP-dependent degradation of Sic1 (Verma et al., 2001). Here, we preincubated proteasomes with PR_20_ at various molar and subsequently followed the time-dependent degradation of Ub-Sic1 or PR_20_.

Ub-Sic1 or PR_20_ levels were determined at 0, 5, and 15 min after exposure to purified 26S proteasomes (Fig. 6*B--D*). In these experiments, we see a time-dependent loss of Ub-Sic1 and when PR_20_ is added to these reactions. There is no change in the abundance of PR_20_ or the alpha 3 subunit of the proteasome over time. Thus, with or without PR_20_, we see specific degradation of Ub-Sic1 in this assay, but PR_20_ is not itself degraded by the proteasome (Fig. 6*B*).

In the absence of PR_20_, there is progressive reduction in Ub-Sic1 abundance, and quantification of signal shows experimental group differences by ANOVA (*F*_(2,9)_ = 773.0, *p* < 0.001). *Post hoc* analysis reveals statistically significantly less Ub-Sic1 at the 5- and 15-min time points in comparison with the starting level (*p* < 0.001 and *p* < 0.001). At a molar ratio of 1:10 or 1:100 (proteasomes:PR_20_), there is also a progressive reduction in Ub-Sic1 abundance and quantification of signal shows experimental group differences by ANOVA (*F*_(2,9)_ = 679.5, *p* < 0.001) and *F*_(2,9)_ = 762.6, *p* < 0.001, respectively). *Post hoc* analysis reveals statistically significantly less Ub-Sic1 at the 5-min (*p* < 0.01 and *p* < 0.01) and 15-min (*p* < 0.01 and *p* < 0.01) time points in comparison with the starting level.

Qualitatively it appears, however, that the progressive reduction of Ub-Sic1 is less pronounced in the presence of PR_20_. To quantify this, we monitored the difference between the Ub-Sic1 signal at the starting point versus the 5- or 15-min time point (e.g., Δ^0–5^ and Δ^0–15^). We then compared the Δ^0–5^ of the three experimental groups (e.g., in the absence of PR_20_, 1:10 proteasomes:PR_20_ and 1:100 proteasomes:PR_20_) and found group differences by ANOVA (*F*_(2,9)_ = 32.42, *p* < 0.001) (Fig. 6*D*). *Post hoc* analysis reveals statistically significant less Δ^0–5^ in the 1:10 and 1:100 experimental groups compared with the no PR_20_ group (*p* < 0.05 and *p* < 0.05). Similarly, we compared the Δ^0–15^ of the three experimental groups and found group differences by ANOVA (*F*_(2,9)_ = 8.667, *p* = 0.008) (Fig. 6*D*). *Post hoc* analysis reveals statistically significantly less Δ^0–15^ in the 1:10 and 1:100 experimental group compared with the no PR_20_ group (*p* < 0.05 and *p* < 0.05). Together, these observations suggest that PR_20_ operates at the level of the proteasome to impede the function of the UPS. This observation is consistent with the hypothesis that PR_20_ causes ubiquitylated substrate build-up due to impaired proteasome function.

### Pharmacological inhibition of the proteasome is toxic to motor neurons, reduces flux of ubiquitylated substrates, and can be rescued by DUB inhibition

**Figure 7.**
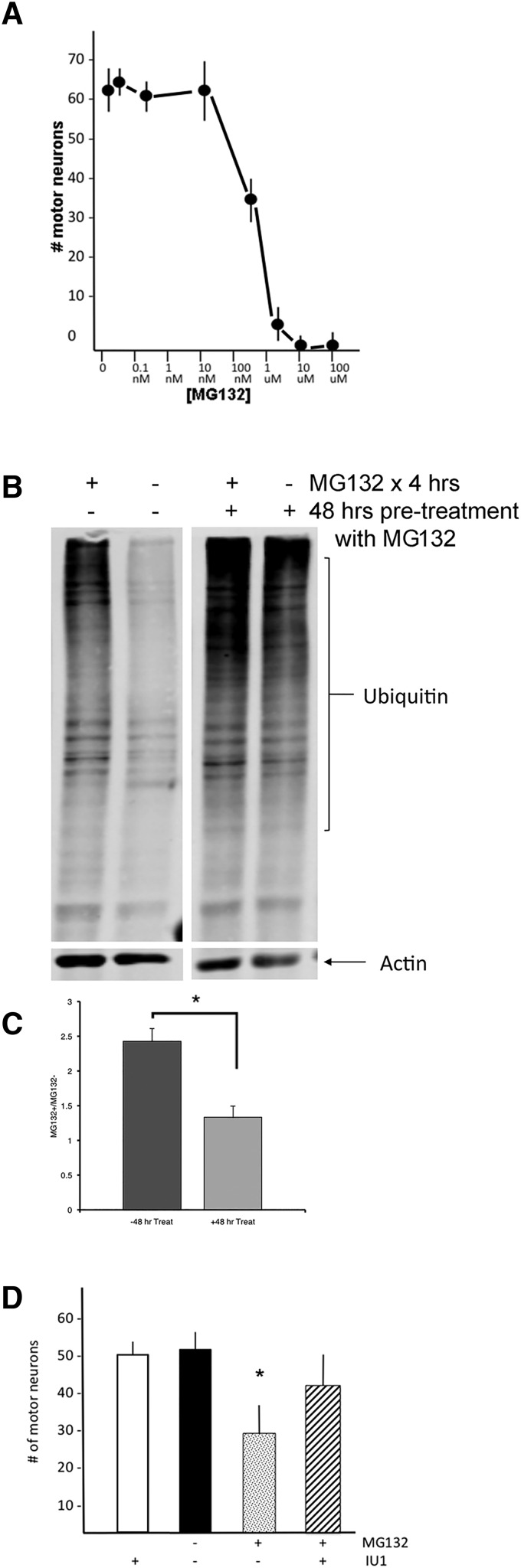
MG132 is toxic to motor neurons, inhibits flux through the proteasome, and cell death is rescued by the DUB inhibitor IU1. MG132 was applied one time at varying concentrations to mixed spinal cord cultures and 5 d later processed for immunocytochemical quantification of motor neurons. ***A***, The dose-response curve shows that MG132 kills motor neurons with an LD50 of ∼ 100-150 nM. ***B***, Mixed spinal cord cultures were exposed to 150 nM MG132 or vehicle for 48 h, and the parallel groups of cultures were exposed to 5 µM MG132 or vehicle for 4 h. Cell lysates were prepared and blotted for total ubiquitin levels. The difference of the ubiquitin immunoreactivity in the cultures treated with 5 µM or vehicle reports the flux of substrates through the UPS over 4 h. Representative blots are shown. ***C***, Quantification of the data from the flux assay and statistical analysis by the permutation tests reveals a statistically significant inhibition of flux through the UPS in cultures treated with 150 nM for 48 h (*p* = 0.027). ***D***, Number of motor neuron in mixed spinal cord cultures after exposure of 150 nM MG132 or vehicle and 5 µM IU1 or vehicle. MG132 leads to a statistically significant reduction in motor neuron number, and this is reversed by IU1 treatment. IU1 alone is not toxic.

If PR_20_ kills motor neurons by partial inhibition of the proteasome, then partial inhibition of the proteasome by pharmacological means might: (1) be toxic to motor neurons and, critically, (2) impair proteasomal flux to a similar degree as seen with PR_20_. These predictions presuppose that proteasomal inhibition by PR_20_ and by MG132 (for example) are equivalent in terms of pharmacodynamics, pharmacokinetics, and selectivity versus nonselectivity in ubiquitylated substrate build-up. We added varying concentrations of MG132 one time to our mixed spinal cord cultures and found an LD_50_ of ∼100-150 nM when motor neuron number was determined 5 d later (Fig. 7*A*). We then studied proteasomal flux by incubating sets of DIV14 cultures with 150 nM MG132 or vehicle for 48 h and then treating half of each set with 5 µM MG132 or vehicle for 4 h before creating cell lysates and probing for ubiquitin (as described above in Figure 4). With *n* = 6 replicates in each experimental group, the permutation test found a statistically significant reduction in proteasomal flux in the 150 nM pretreatment group compared with the control (*p* = 0.027) (Fig. 7*B,C*). Finally, we asked whether proteasomal activation with the USP14 DUB inhibitor IU1 (Lee et al., 2010) could protect motor neurons from MG132 toxicity. Parallel sets of dishes were treated with 150 nM MG132 (or vehicle) and 5 µM IU1 (or vehicle). Counts of motor neurons 5 d later revealed statistically significant group differences by ANOVA (*F*_(8,119)_ = 5.924, *p* < 0.001). The *post hoc* analysis revealed statistically significantly fewer motor neurons in the MG132-treated group in comparison with all other experimental groups (*p* = 0.001). No other group differences were found (Fig. 7*D*). Thus, pharmacological inhibition of the proteasome that achieves a reduction influx equivalent to PR_20_ evokes motor neuron death. This observation supports the view that the toxic activity of PR_20_ could be mediated by proteasomal inhibition; a notion reinforced by the observation that stimulation of the proteasome protects against this level of proteasomal flux inhibition.

### Induction of the proteasome rescues neuronal survival in the presence of PR_20_

**Figure 8.**
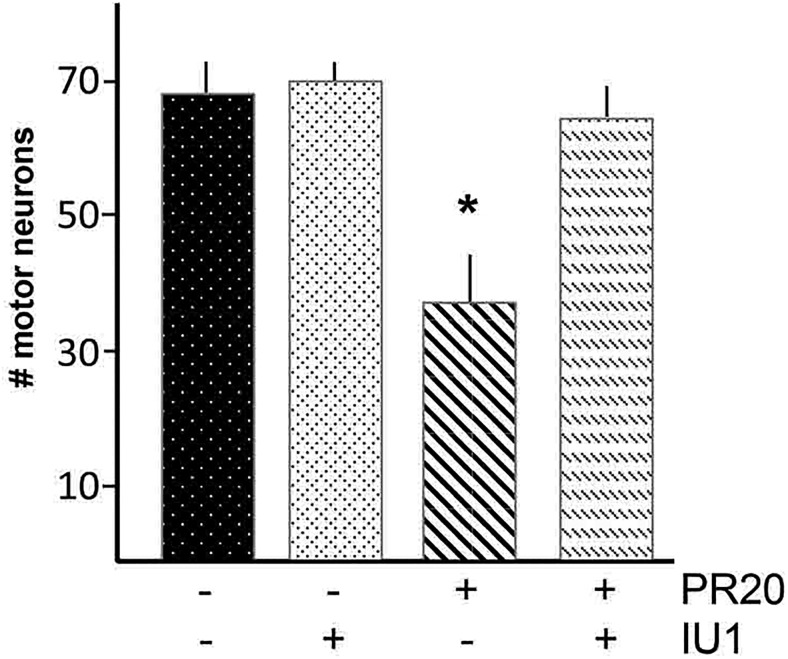
PR_20_-evoked motor neuron death is prevented by the proteasome activator, IU1. Mixed spinal cord cultures were exposed to PR_20_ or vehicle and then dosed with IU1 or vehicle. Motor neuron number was determined 5 d later. In the absence of PR_20_, IU1 did not affect motor neuron survival. PR_20_ treatment leads to approximately 50% loss of motor neurons and IU1 application prevents this effect. n.s., not significant; *, *p* < 0.05.

Finally, we asked whether IU1 protects against PR_20_-induced motor neuron death. We applied PR_20_ (or vehicle) and IU1 (or vehicle) to cultures and quantified the number of motor neurons alive after 5 d of treatment with each condition (Fig. 8). Statistically significant group differences were found by ANOVA (*F*_(8,132)_ = 7.408, *p* < 0.001). The *post hoc* analysis indicated that a statistically significant decrease in motor neuron survival occurs in the presence of PR_20_ and this is rescued to control levels upon treatment with IU1 (*p* < 0.05). IU1 did not influence the survival of vehicle-treated cells. This suggests that the PR_20_-mediated proteasomal degradation dysfunction plays a role in the lethality that the dipeptide imparts on cells.

## Discussion

The mechanism by which *C9ORF72* HRE mutations cause ALS/frontotemporal dementia (FTD) (Renton et al., 2011) is an active area of inquiry. Evidence exists for both a toxic RNA gain-of-function and for the production of toxic DPR proteins (Haeusler et al., 2014; Mizielinska et al., 2014). In an effort to distinguish between the contributions of RNA versus that of DPR proteins, we studied the effects of synthetic dipeptides on mixed cultures. We find that PR_20_ is taken up by neurons and astrocytes and disrupts both the UPS and the autophagic protein degradation pathways. GR_20_, however, did not have an effect on the UPS. PR_20_ has a direct inhibitory effect upon the proteasome when tested *in vitro*. It is likely that these effects of PR_20_ are pathophysiological since pharmacological stimulation of the proteasome promotes motor neuron survival in the presence of PR_20_. The effects of PR_20_ appear molecularly distinct from mTDP43- or mSOD-mediated toxicity (Betz and Hall, 2013; Saxena et al., 2013; Perera et al., 2014; Kong and Xu, 1998). Our results suggest a specific mechanism by which proline/arginine DPR proteins are injurious to motor neurons. Since DPR peptides are seen in human autopsy tissues (Ash et al., 2013; Mori et al., 2013), our *in vitro* observations have implications for human disease.

Among the technical considerations inherent in all disease models, we feel that three merit special discussion. First, many of our experiments are performed after adding PR_20_ to the culture media, achieving an extracellular concentration of 2 µM. What intracellular concentration of PR_20_ does this translate into, does it vary over time and/or by cell-type, and most importantly, how do these values compare to the concentration of cellular DPR proteins generated in other experimental platforms? There are no good data on these difficult questions. It is noteworthy that many investigators have deployed the CMV or CAGG promoters to drive high DPR protein expression from an engineered cDNA (Wen et al., 2014; Zhang et al., 2014; Yamakawa et al., 2015). Unfortunately, we lack the quantitative data on the cellular concentration of RAN peptides in these models that could form the basis of comparison with our results. Second, when using purified proteasomes and a ubiquitylated test substrate *in vitro* (e.g., Ub-Sic1) to determine proteasomal degradation rate, is the PR_20_-dependent inhibition of the proteasome reporting a physiological event? This is a valid concern, since we only see the PR_20_ effect when the molar ratio of PR_20_ to proteasomes is 10:1 or greater. The protein concentration in our *in vitro* reaction is 20 nM, and it is not possible to substantially increase the concentration because the proteins no longer remain soluble. On the other hand, estimates of protein concentrations in cells are about 0.25 g/ml [which for 100-kDa proteins is roughly equivalent to 5 mM (Milo, 2013)], ∼5 orders of magnitude greater than that which can be achieved *in vitro*. The biology of RAN peptides and proteasomes in the crowded environment of cells can, at best, be approximated by *in vitro* assays, although synthetic PR_20_ and purified human proteasomes directly interact with each other. Whether the 10:1 ratio is truly reporting on events occurring *in vitro* will ultimately depend on its predictive value. In light of all of the data we have assembled, we think a reasonable case can be made that the Ub-Sic1/proteasome/PR_20_ observations are valid and relevant. Third, when DPR proteins are expressed from a cDNA, 36 repeat products (Mizielinska et al., 2014; Tran et al., 2015), 42 repeat products (Wen et al., 2014), 50 repeat products (Zhang et al., 2014), 66 repeat products (Kramer et al., 2016), and 100 repeat products (Yamakawa et al., 2015) are toxic. The extent to which cognate cDNAs are translated to generate the intended full-length product is unclear. In addition, the length of DPRs that are benign and the length beyond which toxicity is detectable remains murky. The selection of 20 repeats of PR or GR in our study was based on the observations of others that this length of DPR can be toxic (Kwon et al., 2014; Kanekura et al., 2016). DPRs as short as 6 repeats (such as GR_6_ and GA_6_) can be toxic to neurons; we do not know whether PR_6_ is toxic and if so, whether this involves the proteasome (Flores et al., 2016). Future studies should investigate these issues as well as determine the prevalence of DPRs of specific lengths in human cells with HRE *C9ORF72*.

In an attempt to distinguish the effects of toxic mRNA species from those of DPR proteins, many research groups have taken advantage of the degeneracy in the genetic code to create DPR proteins in the absence of GGGGCC repeats. Whether the cognate mRNA by itself is truly nontoxic remains, in our opinion, an open question. G-C-rich RNAs can adopt a variety of secondary structures even without long, uninterrupted stretches of GGGGCC that could attract and sequester RNA-binding proteins. We believe that studies in which cognate RNA is translated to create DPR proteins do not exclude the possibility of RNA toxicity and thus should be interpreted with caution. Only a few groups have studied the effects of synthetic DPR peptides (unambiguously excluding the contribution of mRNA toxicity in models of *C9ORF72* HRE toxicity) (Kwon et al., 2014; Chang et al., 2016; Kanekura et al., 2016), and only one studied primary neurons (Flores et al., 2016).

Notwithstanding the above concerns using translation of non-GGGGCC constructs to generate diamino acid peptide, many groups have investigated the toxicities of various DPR proteins. Wen et al. (2014) showed that PR_n_ is toxic in primary cortical and motor neuron cultures, and Mizielinska et al. (2014) and Wen et al. (2014) found that PR_n_ and GR_n_, but not PA_n_ and GA_n_, are toxic in a fly model. In primary neuron cultures, GA_n_ and GR_n_ are toxic (May et al., 2014; Zhang et al., 2014; Flores et al., 2016), and this can be associated with ER stress and cytoplasmic/nuclear inclusions. Joviči et al. (2015) also showed PR_n_, PA_n_, and GR_n_ are toxic in a yeast model. Recently described mouse work adds further interpretive difficulties. O’Rourke et al. (2015) showed that GA_n_ and GP_n_ dipeptides accumulated in a bacterial artificial chromosome transgenic mouse model but were not associated with phenotypic changes to mouse behavior or neurodegeneration. On the other hand, Yamakawa et al. (2015) found GA_n_ linked behavioral deficits using *in utero* electroporation in mice to express the dipeptides in the cerebral cortex. In sum, while there is no question that DPR proteins are present in patients and in various disease models, precisely which species are toxic (or the extent to which they are toxic) remains contentious.

We, and others (Kwon et al., 2014), have found that PR_20_ accumulates in the nucleus, raising the interesting prospect that inhibition of nuclear proteasomes underlies the pathophysiology. Protein quality control (PQC) pathways manage misfolded proteins within distinct subcellular compartments (Shibata and Morimoto, 2014). There is a rich literature about PQC in the ER and cytoplasm, but less is known about PQC in the nucleus. The UPS is the main mechanism for protein degradation in the nucleus (Jones and Gardner, 2016; Gallagher et al., 2014; Shibata and Morimoto, 2014) and the nucleus harbors the majority of cellular proteasomes (Wójcik and DeMartino, 2003; although see Dang et al., 2016). In yeast, the San1 ubiquitin ligase targets misfolded nuclear proteins for proteasomal degradation (Gardner et al., 2005; Rosenbaum et al., 2011). Other ubiquitin ligases implicated in nuclear PQC include Ubr1, Doa10, and Asi, others are likely to exist (Jones and Gardner, 2016). In addition to targeting misfolded nuclear proteins, some cytosolic proteins (i.e., polyQ-expanded proteins) are imported into the nucleus for degradation (Park et al., 2013). The features of damaged cytosolic proteins that identify them for nuclear import and disposal by the nuclear PQC pathway are unknown. PR_20_ inhibition of nuclear proteasomes could have broad effects not only on the integrity of the nuclear proteome but also on interconnected PQC pathways in other subcellular domains.

In various disease model platforms, HRE RNA foci and DPR protein puncta have been demonstrated. Our understanding of how these entities are linked to cell dysfunction and death is inchoate. Additionally, defects in nucleocytoplasmic shuttling, ER stress, proteasomal dysfunction, and nucleolar stress have also been described in various models. However, a detailed connection of these processes gone awry and disease pathogenesis is not well understood (Kwon et al., 2014; Wen et al., 2014; Freibaum et al., 2015; Joviči et al., 2015; Yamakawa et al., 2015; Zhang et al., 2015; Zhang et al., 2016).

Here, we show that PR_20_ dipeptides kill motor neurons and lead to a time-dependent build-up of ubiquitylated substrates, an effect we ascribed to an impairment of both the UPS and autophagy pathways. PR_20_ physically associated with the proteasome and we suggest that this interaction underlies the functional impairment of the UPS. That another RAN translation product, GR_20_, is neither toxic to motor neurons nor leads to impairment in UPS flux, highlights the specificity of the proposed molecular mechanism for PR_20_ toxicity. To understand why the accumulation of ubiquitylated substrates is toxic, it will be important to determine which ubiquitylated substrates accumulate in the presence of PR_20_. Two broad possibilities exist: (1) PR_20_ may nonspecifically inhibit the degradation of ubiquitylated substrates, and this could be toxic owing to the accumulation of damaged/misfolded proteins; or (2) PR_20_ may inhibit the degradation of a specific subset of ubiquitylated substrates, and this could be toxic owing to the disruption of a select cell biological process. Resolving the contributions of these alternative possibilities may provide mechanistic insight into the pathophysiology.

## Synthesis

Reviewing Editor: Jörg Schulz, RWTH Aachen University Decisions are customarily a result of the Reviewing Editor and the peer reviewers coming together and discussing their recommendations until a consensus is reached. When revisions are invited, a fact-based synthesis statement explaining their decision and outlining what is needed to prepare a revision will be listed below. The following reviewer(s) agreed to reveal their identity: Steven Finkbeiner, Sebastian Grönke.

The authors addressed all criticism that was raised.
